# Obstipation

**Published:** 1901-08-10

**Authors:** 


### OBSTIPATION.

Obstipation is a term which has so far hardly found a home in English medical literature, but it is largely used in America, and according to certain specialists in diseases of the rectum the condition which the term describes, namely obstructive costiveness, is far more common and much more important than many medical men have any idea of. Dr. Sterling B. Taylor,[1] "Proctologist to the Grant Hospital, Columbus," says that the type of costiveness which has received the name of obstipation is dependent upon some mechanical or foreign interference situated in the rectum or signoid flexure. In constipation there is a sluggishness of peristalsis which delays bowel movement, but eventually there is an effective and satisfactory effort at defæcation. In obstipation, however, the condition is the reverse. There is a constant desire which is only partially relieved by physic and enemata. These produce a stool which is either liquid, semi-liquid, or tapelike in form, but there is still left an uncontrollable desire for further and more complete defæcation. The causes of this condition are (1) compression from without; (2) blocking of the lumen of the canal; (3) constriction arising from disease of the gut wall; (4) hypertrophy of the normal rectal valves with consequent interruption of the lumen of the canal and accompanying distortion of the gut above the obstruction, the latter begetting an atonic state. The first three of these causes are matters of common knowledge. It is to the fourth condition, namely hypertrophy of the rectal valves, that so much attention has of late been paid in America, and to which the "proctologists" more particularly apply the term obstipation. The rectal valve, we are told, spans half the diameter of the rectum, is semilunar in shape, and must be considered as an anatomical entity in studying the mechanism of defæcation. As a rule there are three of these valves, but there may be more or fewer, and continually exposed as they are to violence, it is only to be expected that they should often exhibit pathological changse, besides which they are no doubt liable to congenital malformations. One only needs to see a demonstration of the normal valve, we are told, and then to see one that has been thickened and enlarged, to understand how these valves may not only induce constipation but actually become the obstructive agent in obstipation.

Possibly the office of the normal valve is to support and retard the descent of the fæcal mass, but it can be shown experimentally that when hypertrophied it can support matter placed on its upper surface, and that even when the valve is pulled down by a hook it is not effaced, while after section of the valve has been made any such body will be retained above it with great difficulty. One hypertrophied valve may cause all the trouble, but all may be affected. By taking a complete history of the case one may arrive at a presumptive diagnosis that we have to do with a case of obstructive constipation, but it is only by physical examination that we can eliminate the other causes of obstruction and ascertain the existence of hypertophied valves. We are told these valves may be very difficult to discover unless they are sought for in a very special manner, and, as we read between the lines, by a somewhat special man. One has not merely to discover the valves but to decide how far they, and how far the consequent "angulation of the gut due to dilatation," are the real cause of the constipation. Having previously made a preliminary examination to ascertain that the proctoscope is admissible, the patient should be placed in the "Martin's position"—a modified knee-chest position—the obturator of the warmed proctoscope should be placed against the anus, the patient be told to bear down, and then the instrument must be inserted. When the obturator is withdrawn the rectum will at once balloon, exposing its entire mucous surface from the first valve. Each valve should then be successively exposed and tested. The treatment may be palliative in the shape of massage, enemas, diet, etc., or valvotomy may be performed. In some cases it is said the band offers almost as much resistance to cutting as cartilage would. After division of the free border the incision should be carefully continued down almost to the gut wall. Sutures should be placed one in the angle of the wound on each side. A tampon may or may not be introduced as a further guard against hæmorrhage. The after-treatment includes daily lavage; and finally we are told that this operation is "one of the most delicate in surgery," and one that should not be undertaken by anyone without a thorough knowledge of its technique.

[1] New York Med. News, June 22.

